# Association between blood cadmium levels and the risk of osteopenia and osteoporosis in Korean post-menopausal women

**DOI:** 10.1007/s11657-021-00887-9

**Published:** 2021-02-02

**Authors:** Eun-San Kim, Sangah Shin, Yoon Jae Lee, In-Hyuk Ha

**Affiliations:** 1 Jaseng Spine and Joint Research Institute, Jaseng Medical Foundation, 3F, 538 Gangnam-daero, Gangnam-gu, Seoul, 06110 Republic of Korea; 2 Department of Food and Nutrition, Chung-Ang University, Gyeonggi-do, 17546 Republic of Korea

**Keywords:** Cadmium, Osteoporosis, Osteopenia, Post-menopausal women

## Abstract

***Summary*:**

We aimed to investigate the association between cadmium levels and the risk of osteopenia and osteoporosis in Korean post-menopausal women. There was a significant positive association between cadmium levels and the risk of osteopenia and osteoporosis, but further studies for dose response are required.

**Purpose:**

Cadmium exposure can exert detrimental effects on bone health, particularly in post-menopausal women. However, previous studies have failed to report an association in Korean post-menopausal women. We aimed to investigate the association between cadmium levels and the risk of osteopenia and osteoporosis in Korean post-menopausal women.

**Methods:**

In total, 5432 participants from the 4th and 5th Korean National Health and Nutrition Examination Survey (KNHANES) were randomly sampled for measurements of heavy metal concentrations in the blood, bone mass density (BMD), and nutrient intake. We analyzed data for 1031 post-menopausal women ≥50 years of age. Blood cadmium levels were categorized into quartiles, and a multinomial logistic regression model was used for analysis.

**Results:**

There was a significant positive association between cadmium levels and the risk of osteopenia and osteoporosis, but the odds ratio (OR) at the 4th level was lower than that at the 3rd level (OR and 95% confidence interval (CI) for osteopenia: 2nd quartile: 1.24, 0.88-1.74; 3rd quartile: 3.22, 2.24-4.64; 4th quartile: 1.27, 0.87-1.85; *P* for trend <0.001; OR and 95% CI for osteoporosis: 2nd quartile: 1.54, 1.05-2.25; 3rd quartile: 3.63, 2.31-5.69; 4th quartile: 1.70, 1.03-2.81; *P* for trend <0.001). This trend was consistent in the sensitivity analysis.

**Conclusion:**

Our findings suggest that there is an association between blood cadmium levels and the risk of osteopenia and osteoporosis in Korean post-menopausal women. However, further prospective studies are required to determine whether there is a dose-response relationship and address potential selection bias, especially in patients with femoral neck osteoporosis.

**Supplementary Information:**

The online version contains supplementary material available at 10.1007/s11657-021-00887-9.

## Introduction

Cadmium exposure is a major risk factor for osteoporosis. Cadmium can accumulate in the body via occupation-related exposure, smoking, diet, and other sources [1]. Cadmium disturbs the mineralization of bone by damaging the renal tubular system, interfering with parathyroid hormone (PTH) and vitamin D metabolism, reducing absorption of calcium from the intestine, and directly damaging the bone [2, 3]. The risk of cadmium exposure has been identified in various populations [4–6], including post-menopausal women [7, 8], who are particularly vulnerable to osteoporosis [9].

However, previous studies have failed to identify a positive association between cadmium exposure and osteoporosis in Korean post-menopausal women [10]. We attempted to supplement this work using various methods: More samples were included to reduce random bias, multiple imputations were used for missing data to reduce selection bias, the weights were adjusted given the complex survey design [11], a multinomial model was used for precise estimation of associations [6], and the model was constructed in consideration of exposure, confounders, and outcomes [12]. Therefore, in the present study, we aimed to investigate the association between cadmium exposure and the risk of osteopenia and osteoporosis in Korean post-menopausal women to address the remaining gaps in knowledge.

## Methods

### Participants

The present study utilized samples from the 4th and 5th Korean National Health and Nutrition Examination Survey (KNHANES). The original data were obtained from July 2008 to May 2011. Heavy metal levels, bone mass density (BMD), general health, and nutrition were assessed during this period. The KNHANES is a nationwide cross-sectional survey conducted each year by the Korea Centers for Disease Control and Prevention. The goal of the KNHANES is to assess and monitor health and nutritional status in the Republic of Korea. The target population consists of all non-institutionalized citizens residing in Korea. The KNHANES utilizes a complex survey design and is constructed via two-stage stratified cluster sampling. The KNHANES represents the target population using sampling weights. Additional details have been described in a previous study [13].

**Fig. 1:**
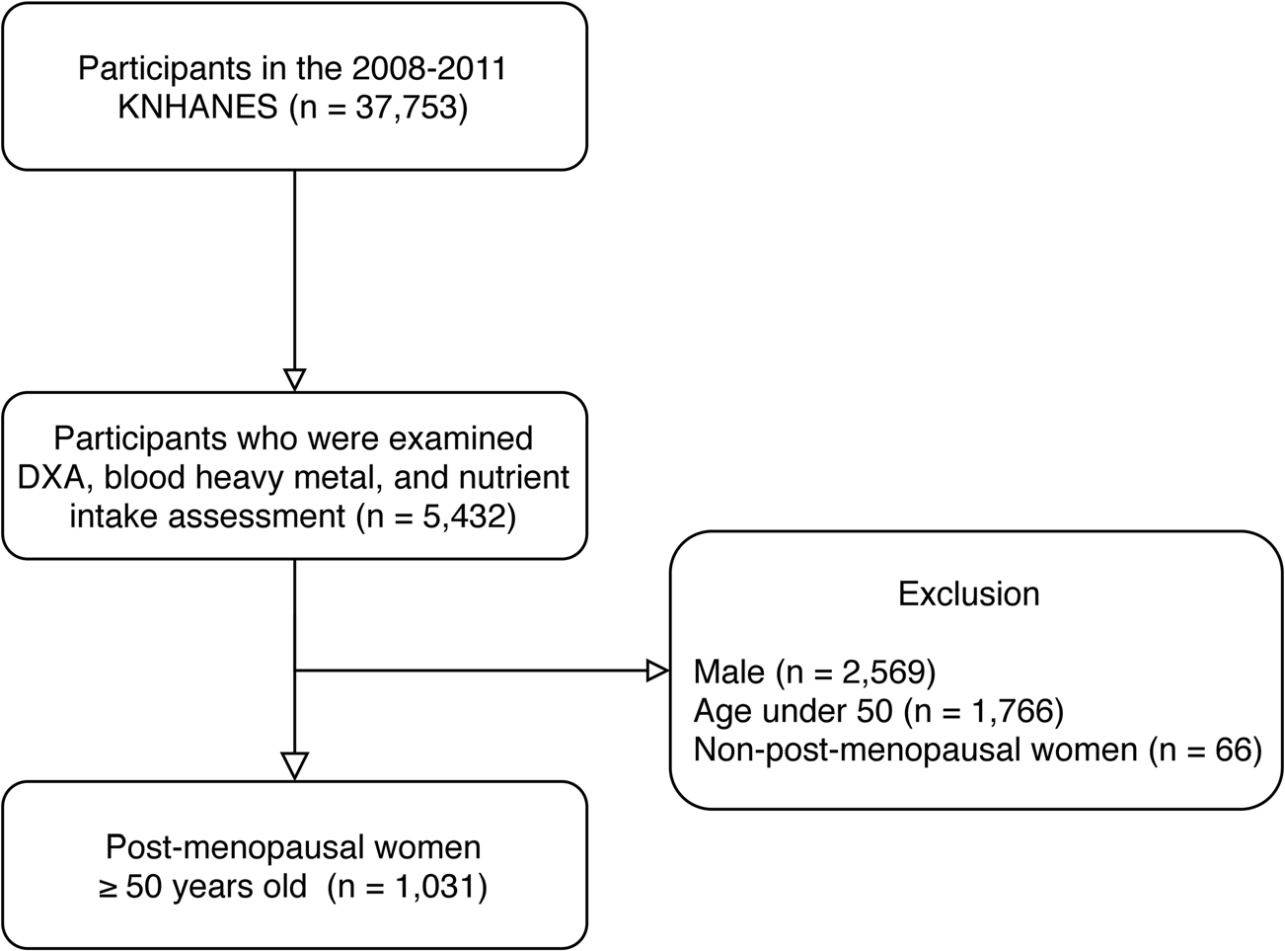
Flowchart of participant selection

The population of the present study consisted of post-menopausal women ≥50 years of age. Among 37,753 participants in the 2008-2011 KNHANES, a total of 5432 participants were randomly sampled for measurements of heavy metal concentrations in the blood, BMD, and nutrient intake. Male participants (*n* = 2569), those under age 50 (*n* = 1766), and non-post-menopausal women (*n* = 66) were excluded from the analysis. Multiple imputations were then used for missing covariates in the remaining 1031 participants (Fig. 1). Other exclusion criteria (e.g., early menopause, cancer) were not adopted, as these variables are effect modifiers or mediators [14] rather than confounders in the present model [15, 16] (Online Resource 1).

### Blood cadmium levels

Blood samples were obtained from participants for the measurement of blood cadmium levels. Samples were collected in vacuum tubes (vacutainer), mixed with anticoagulants, and stored in a refrigerator. They were analyzed within 24 h in the diagnostic laboratory of NeoDin Medical Institute in Seoul, South Korea. Blood levels of cadmium were measured via graphite furnace atomic absorption spectrometry (GFAAS) using a Perkin Elmer AAnalyst 600 system (PerkinElmer, Turku, Finland). In the internal validation step, inter-assay coefficients of variation (CVs) continued to be within an acceptable range (≤10%). External validation was performed by the Korea Occupational Safety and Health Agency and German External Quality Assessment Scheme (G-EQUAS). The benchmark dose of blood cadmium has yet to be determined for the Korean post-menopausal population. Therefore, blood cadmium levels were categorized into quartiles following previous study [10].

### BMD, osteopenia, and osteoporosis

BMD was measured for the total hip, femoral neck, and lumbar spine (L1–L4) using dual-energy X-ray absorptiometry (DXA, DISCOVERY-W fan-beam densitometer; Hologic Inc., Bedford, MA, USA). For precision assessment, radiologic technologists were trained to make the CVs of randomly double-checked participants ≤1.8% for the total hip, ≤2.5% for the femoral neck, and ≤1.9% for the lumbar spine. Osteoporosis and osteopenia were defined according to T-scores. The T-score is the standard deviation from the average peak BMD for sex-matched and race-matched (Japanese) populations [17]. Osteoporosis was defined as a T-score of −2.5 or less at any site, while osteopenia was defined as a T-score less than −1.0 and greater than −2.5.

### Confounders

To minimize confounding bias, we selected confounders based on the disjunctive cause criterion. In this criterion, variables that are cause of exposure, outcome, or both are controlled. If exposure is the cause of a variable, it is regarded as a mediator or collider, rather than a confounder. Adjusting these variables would lead to bias [18]. A detailed explanation of confounder selection is presented in the form of a directed acyclic graph (DAG) (Online Resource 1).

Age, education level, household income (quartile), occupation, and residence were included as confounders reflecting socioeconomic status [1, 19]. To minimize residual confounding, age was adjusted as both a continuous and categorical variable. The year in which participants were surveyed was also adjusted [20].

Body mass index (BMI) was calculated based on measured height and weight [21]. Lifestyle factors such as current alcohol consumption, smoking exposure, and physical activity were also included [1, 19, 22]. When defining smoking exposure, we considered that women smokers tend to under-report their smoking status by more than 50%, as identified by a previous KNHANES [23]. Therefore, we defined smoking exposure as 50 ng/mL or more urinary cotinine, based on the results of previous studies [24, 25]. Gas chromatography-mass spectrometry (GC-MS) with a Clarus 600/600 T system (Perkin Elmer, Waltham, MA, USA) was used to measure urinary cotinine. Other information was obtained using questionnaires. Alcohol consumption was categorized into “less than once per month,” “greater than once per month but less than twice per week,” and “greater than twice per week” [26]. Physical activity was categorized as vigorous (3 days/week, ≥20 min/session), moderate (5 days/week, ≥30 min/session), or daily walking (5 days/week, ≥30 min/session).

Vitamin D levels were measured based on serum 25-hydroxyvitamin D(25(OH)D3) concentration using a radioimmunoassay (1470 WIZARD gamma-Counter, PerkinElmer, Turku, Finland) [27]. Food consumptions were recorded based on the 24-h dietary recall method, following which nutrient intake was calculated. Fish and seaweed consumption [28] as well as energy, protein, calcium, phosphate, potassium, and retinol intake [29] were included in the model. Intake of certain foods was classified as less than once a week, once a week, or more than once a week [30]. Nutrient intake was adjusted as a continuous variable. The previous use of hormone therapy or oral contraceptive (>1 month of use) was obtained using a questionnaire. The use of hormone therapy was categorized into “never,” “less than 2 years,” and “2 years or more” [31, 32].

### Statistical analysis

Among the study population (*n* = 1031), 122 participants (11.8%) had at least one missing covariate or outcome value. Multiple imputations were used to impute item non-response. Assuming missing at random (MAR), all variables in the model were included in the multiple imputations. For continuous variables, we used predictive mean matching due to their non-normal distribution. For categorical variables, we used different types of logistic regression for categorical variables based on the variable type (i.e., binary, multinomial, or ordinal). Imputation was performed using the Markov chain Monte Carlo (MCMC) method, and 20 imputed sets were created.

The design and sampling weights of all participants in the KNHANES were included in the analysis to account for the complex survey design. Multinomial logistic regression was used to analyze the associations between cadmium and the risk of osteopenia and osteoporosis. Blood cadmium levels were divided into quartiles, as previously described [10]. In the multinomial model, we used replicate weights with the bootstrap method to estimate 95% confidence intervals (CIs). Bootstrapping was performed 250 times with five imputed sets each. Regression methods were used to examine linear *P* trends in blood cadmium levels.

### Exploratory and sensitivity analyses

For exploratory purposes, we investigated the association of cadmium with osteopenia and osteoporosis by subregion. Osteopenia and osteoporosis in the total hip, femoral neck, and lumbar spine were defined according to T-scores. The multinomial model was respectively used in each subregion except for osteoporosis in total hip due to the low number of cases.

Sensitivity analyses were performed based on three different outcome definitions. (1) Some patients may undergo treatment following the development of osteoporosis, leading to an increase in T-score. If the proportion of treated patients varies based on cadmium levels, using only current T-scores to define osteoporosis can lead to biased estimates. Therefore, patients with T-scores greater than −2.5 answering “yes” to “Have you ever been diagnosed with osteoporosis by a doctor?” and “Are you currently under treatment for osteoporosis?” were classified into the osteoporosis group. (2) The reference for calculating T-scores was derived from a Japanese population [17]; however, reference values may differ for Korean and Japanese populations [33]. Therefore, osteopenia and osteoporosis were defined according to T-scores that had been re-calculated using Korean reference values. (3) T-scores based on Korean reference values and current treatment information were both used to define osteoporosis and osteopenia.

To examine the robustness of the results, additional sensitivity analysis was performed. (1) Blood cadmium level was categorized into quintile (five sections). (2) Outcome was defined as T-score −1.8 or less and −2.0 or less. For each outcome, logistic regression was used. (3) BMD and T-score at each site were analyzed. Moreover, the lowest BMD and T-score among sites were analyzed.

### Ethical approval

The study protocol was approved by the Institutional Review Board of Jaseng Hospital of Korean Medicine (JASENG 2020-08-019), who waived the requirement for informed consent due to the nature of the study.

## Results

The general characteristics of the participants are presented in Table 1. Mean blood cadmium levels were 0.81 ± 0.02 (mean ± SE) in the 1st quartile and 2.43 ± 0.05 (mean ± SE) in the 4th quartile. Level of education, household income, phosphate intake, and retinol intake decreased as cadmium levels increased. Moreover, the prevalence of smoking exposure was higher in the 3rd and 4th quartiles than in the 1st and 2nd quartiles.

**Table 1:** Basic characteristics of the study population

	Blood cadmium levels			
	First quartile	Second quartile	Third quartile	Fourth quartile
	(*n* = 258)	(*n* = 258)	(*n* = 258)	(*n* = 257)
Blood cadmium level (μg/L)	0.81 ± 0.02	1.23 ± 0.01	1.59 ± 0.01	2.43 ± 0.05
Age in years	63.3 ± 0.7	63.9 ± 0.8	63.0 ± 0.8	63.7 ± 0.8
Age by group (%)				
50-59 years	39.1	37.8	41.6	40.7
60-69 years	30.1	28.1	32.5	28.1
≥70 years	30.9	34.1	25.9	31.2
Highest education (%)				
Elementary school or no schooling	58.2	59.7	62.3	74.4
Middle school	14.1	20.9	16.6	12.5
High school	27.7	19.5	21.2	13.1
Household income (%)				
1st quartile	37.4	37.9	34.4	42.7
2nd quartile	23.3	23.9	27.5	27.5
3rd quartile	20.9	24.2	17.1	17.7
4th quartile	18.5	14.0	21.0	12.1
Occupation (%)				
Agriculture	9.5	11.1	7.7	9.8
Engineering/mechanical work	16.5	16.1	12.6	11.4
Others	10.3	13.2	15.6	13.3
None	63.7	59.6	64.1	65.4
Residence (%)				
Urban	74.2	74.2	80.2	75.2
Rural	25.8	25.8	19.8	24.8
Year (%)				
2008	22.0	13.3	17.0	16.8
2009	36.0	31.3	33.9	34.5
2010	28.5	37.5	35.4	33.8
2011	13.6	18.0	13.7	14.9
Current drinker (%)				
Less than once per month	79.5	78.4	75.0	78.8
Greater than once per month but lesser than twice per week	17.3	16.8	18.8	15.6
Greater than twice per month	3.2	4.8	6.2	5.5
Smoking exposure (%)	3.2	4.8	8.9	8.3
Vigorous-intensity physical activity (%)	16.4	11.3	15.0	15.9
Moderate-intensity physical activity (%)	15.3	11.1	17.7	10.9
Daily walking (%)	48.3	35.4	40.8	35.8
Body mass index (kg/m^2^)	24.3 ± 0.2	24.4 ± 0.3	24.1 ± 0.2	24.7 ± 0.3
Serum 25-hydroxyvitamin D (ng/mL)	19.3 ± 0.6	17.3 ± 0.6	18.2 ± 0.7	17.2 ± 0.6
Fish consumption (%)				
Less than once a week	25.2	18.1	25.1	30.8
Once a week	23.2	20.4	17.6	19.2
Over once a week	51.6	61.4	57.2	50
Seaweed consumption (%)				
Less than once a week	31.4	18.8	22.6	32.4
Once a week	20.4	20.1	24.8	17.9
Over once a week	48.2	61.2	52.5	49.7
Energy intake per day (Kcal)	1571.0 ± 42.1	1549.0 ± 51.4	1519.9 ± 44.2	1551.2 ± 47.7
Protein intake per day (g)	53.8 ± 2.0	50.9 ± 2.1	51.7 ± 2.2	49.2 ± 1.9
Calcium intake per day (mg)	434.9 ± 22.1	383.4 ± 18.6	455.0 ± 32.6	380.9 ± 20.1
Phosphate intake per day (mg)	983.0 ± 31.5	934.2 ± 33.3	932.8 ± 37.2	916.7 ± 32.5
Potassium intake per day (mg)	2753.7 ± 106.8	2535.1 ± 107.7	2486.1 ± 98.9	2620.8 ± 128.4
Retinol intake per day (μg)	94.9 ± 23.7	51.0 ± 5.4	53.3 ± 6.5	44.2 ± 7.1
Oral contraceptive use (%)	21.7	23.6	29.8	26.3
Hormone replacement therapy (%)				
Never	82.2	73.8	84.9	90.1
Less than 2 years	8.5	12.8	9.1	5.8
2 years or more	9.4	13.4	6.0	4.1

Cadmium exposure was divided into quartiles. The cut-off points for quartiles were 1.04 μg/L, 1.40 μg/L, and 1.82 μg/L (1.01 μg/L, 1.36 μg/L, 1.78 μg/L in weighted value for complex survey design). The basic characteristics are presented as the mean ± standard error of the mean for continuous variables and as percentages for categorical variables

The prevalence rates and odds ratios (ORs) for osteoporosis and osteopenia are presented according to cadmium levels in Table 2. Higher cadmium levels were significantly associated with both osteopenia and osteoporosis, except the OR for osteopenia in the 4th quartile. The ORs for osteopenia and osteoporosis decreased at the 4th quartile but the *P* trend indicated significant positive association. ORs were generally higher for osteoporosis than for osteopenia (OR and 95% CI for osteopenia, 2nd quartile: 1.24, 0.88-1.74; 3rd quartile: 3.22, 2.24-4.64; 4th quartile: 1.27, 0.87-1.85; *P* for trend <0.001; for osteoporosis, 2nd quartile: 1.54, 1.05-2.25; 3rd quartile: 3.63, 2.31-5.69; 4th quartile: 1.70, 1.03-2.81; *P* for trend < 0.001).

**Table 2:** Association between blood cadmium levels and the risk of osteopenia and osteoporosis

	Osteopenia			Osteoporosis		
BCd level	Prevalence	Unadjusted	Adjusted	Prevalence	Unadjusted	Adjusted
First quartile	50.55 (42.42-58.67)	Ref	Ref	33.90 (26.21-41.58)	Ref	Ref
Second quartile	47.64 (39.34-55.94)	1.06 (0.85-1.32)	1.24 (0.88-1.74)	38.33 (29.86-46.80)	1.26 (0.98-1.63)	1.54 (1.05-2.25)
Third quartile	55.94 (47.60-64.28)	2.16 (1.64-2.85)	3.22 (2.24-4.64)	36.06 (27.90-44.22)	2.07 (1.58-2.73)	3.63 (2.31-5.69)
Fourth quartile	45.25 (36.76-53.74)	1.05 (0.82-1.34)	1.27 (0.87-1.85)	41.47 (32.41-50.53)	1.42 (1.10-1.84)	1.70 (1.03-2.81)
*P* for trend	━	━	< 0.001	━	━	< 0.001

The association between blood cadmium levels and the risk of osteopenia and osteoporosis was analyzed using a multinomial logistic regression model. Osteopenia and osteoporosis were defined based on T-scores. The prevalence rates and odds ratios (ORs) are presented with 95% confidence intervals for each quartile. BCd: blood cadmium

**Fig. 2:**
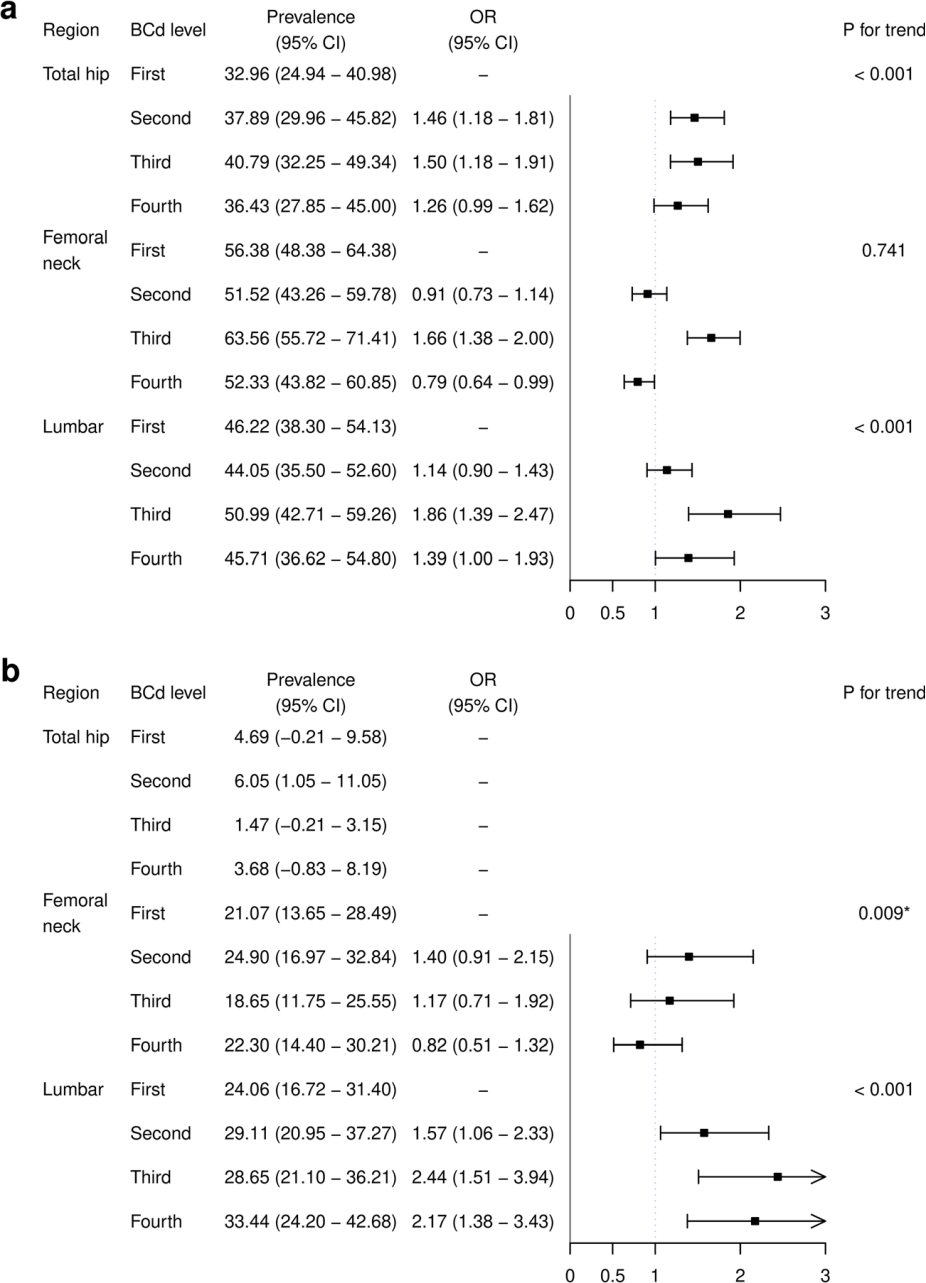
Secondary analysis of associations between blood cadmium levels and the risk of osteopenia and osteoporosis by subregion. We performed a secondary analysis to investigate the association between cadmium levels and the risk of osteopenia and osteoporosis by subregion. Osteopenia and osteoporosis in the total hip, femoral neck, and lumbar spine were defined according to T-scores (osteoporosis: −2.5 or less; osteopenia: less than −1.0 and greater than −2.5). A multinomial logistic regression model was used to assess osteopenia and osteoporosis in each subregion. The prevalence rates and odds ratios (ORs) are presented with 95% confidence intervals (CIs) for each quartile. * The *P* trend for osteoporosis of the femoral neck was significant, but the association was negative. BCd: blood cadmium

The trend of association was generally consistent in the exploratory and sensitivity analyses. However, when the exploratory analysis was performed by subregion (Fig. 2), the OR for the femoral neck in the 4th quartile was significantly lower than 1 for osteopenia. Moreover, the *P* trend indicated a significant negative association for osteoporosis in the femoral neck (OR in the 4th quartile and 95% CI for osteopenia of femoral neck: 0.79, 0.64-0.99; *P* for trend of osteoporosis in femoral neck: 0.009).

In the first sensitivity analysis, which considered current treatment in the outcome definition, the ORs for osteoporosis slightly increased in the 3rd and 4th quartiles (OR and 95% CI for osteoporosis, 3rd quartile: 3.90, 2.46-6.16; 4th quartile: 1.85, 1.14-2.99). However, when T-scores were calculated using Korean reference values, the ORs generally decreased, and only the ORs for osteoporosis in the 3rd quartile remained significant (OR and 95% CI for osteoporosis, 3rd quartile: 1.80, 1.16-2.81). When current treatment and Korean reference values were both considered, the ORs for osteoporosis increased and 4th quartile became significant (OR and 95% CI for osteoporosis: 3rd quartile: 1.92, 1.39-2.67, 4th quartile: 1.72, 1.10-2.69). The *P* trend indicated significant positive association in all models (Table 3).

**Table 3:** Sensitivity analysis for different outcome definitions of osteopenia and osteoporosis

	Osteopenia			Osteoporosis		
BCd level	Prevalence	Unadjusted	Adjusted	Prevalence	Unadjusted	Adjusted
Model 1						
First quartile	46.82 (38.75-54.89)	Ref	Ref	37.62 (29.87-45.38)	Ref	Ref
Second quartile	43.28 (35.10-51.46)	0.99 (0.77-1.28)	1.24 (0.90-1.69)	42.69 (34.23-51.15)	1.25 (0.99-1.58)	1.61 (1.10-2.37)
Third quartile	49.61 (41.25-57.97)	2.10 (1.54-2.86)	3.14 (2.14-4.60)	42.39 (33.97-50.81)	2.23 (1.69-2.96)	3.90 (2.46-6.16)
Fourth quartile	41.56 (33.48-49.64)	1.08 (0.81-1.45)	1.34 (0.88-2.03)	45.83 (36.95-54.71)	1.47 (1.12-1.94)	1.85 (1.14-2.99)
*P* for trend	━	━	< 0.001	━	━	< 0.001
Model 2						
First quartile	48.69 (40.39-56.99)	Ref	Ref	29.75 (22.15-37.34)	Ref	Ref
Second quartile	51.85 (43.10-60.59)	1.11 (0.91-1.36)	1.13 (0.90-1.41)	27.46 (19.32-35.59)	0.95 (0.74-1.21)	0.98 (0.67-1.43)
Third quartile	53.65 (45.25-62.06)	1.45 (1.21-1.75)	1.66 (1.30-2.11)	29.95 (22.06-37.85)	1.29 (1.04-1.59)	1.80 (1.16-2.81)
Fourth quartile	47.22 (38.68-55.76)	1.16 (0.96-1.40)	1.25 (0.92-1.68)	34.78 (25.58-43.97)	1.38 (1.06-1.78)	1.50 (0.95-2.38)
*P* for trend	━	━	< 0.001	━	━	< 0.001
Model 3						
First quartile	44.94 (36.71-53.18)	Ref	Ref	33.49 (25.79-41.19)	Ref	Ref
Second quartile	44.91 (36.16-53.66)	1.02 (0.85-1.23)	1.06 (0.83-1.36)	34.40 (25.88-42.91)	1.05 (0.85-1.30)	1.13 (0.79-1.60)
Third quartile	48.22 (39.83-56.61)	1.48 (1.18-1.84)	1.69 (1.30-2.19)	36.02 (27.63-44.41)	1.48 (1.19-1.83)	1.92 (1.39-2.67)
Fourth quartile	41.22 (33.11-49.33)	1.14 (0.92-1.42)	1.20 (0.86-1.65)	41.45 (32.33-50.57)	1.56 (1.20-2.02)	1.72 (1.10-2.69)
*P* for trend	━	━	< 0.001	━	━	< 0.001

Model 1: T-score criteria (osteoporosis: −2.5 or less; osteopenia: less than −1.0 and greater than −2.5) and current treatment information (patients with T-scores greater than −2.5 but currently undergoing osteoporosis treatment were classified as having osteoporosis). Model 2: T-score criteria calculated using reference values for the Korean population. Model 3: T-score criteria calculated using Korean reference values and current treatment information. The prevalence rates and odds ratios (ORs) are presented with 95% confidence intervals for each quartile. BCd: blood cadmium

In the analysis performed with the quintile, the OR in the 3rd and 4th quintile was significant for osteopenia and osteoporosis, but the OR decreased in the 5th quintile and became non-significant (OR and 95% CI for osteopenia, 3rd quintile: 2.45, 1.49-4.01; 4th quintile: 1.69, 1.02-2.82; 5th quintile: 1.20, 0.75-1.93; *P* for trend <0.001; for osteoporosis, 3rd quartile: 2.42, 1.31-4.49; 4th quartile: 2.83, 1.48-5.40; 5th quartile: 1.66, 0.86-3.19; *P* for trend < 0.001) (Online Resource 2). An analysis with T-score −2.0 or less showed significant OR in the 4th quartile and the trend showed dose response (OR and 95% CI for T-score −2.0 or less, 4th quartile: 1.76, 1.02-3.05; *P* for trend: 0.027) (Online Resource 3). When BMD and T-score were analyzed as outcomes, the 3rd quartile was significant only for lowest T-score among sites (estimates and 95% CI for lowest T-score, 3rd quartile: −0.17, −0.34 to −0.01) (Online Resource 4).

## Discussion

Our findings indicated that the risks of osteopenia and osteoporosis increased as blood cadmium levels increased. However, the OR at the highest (4th quartile) blood cadmium level was lower than that at the 3rd quartile, despite a significant *P* trend, making the dose-response relationship somewhat unclear. This trend was consistent with that observed in the sensitivity analysis performed using Korean reference values for T-scores. Thus, although we concluded that blood cadmium levels affect the risk of osteoporosis and osteopenia in post-menopausal women, our findings should be interpreted with caution.

There are some explanations for the absence of a dose-response relationship at the highest cadmium levels. First, when current treatment information was considered in the definition of osteoporosis, the OR for osteoporosis at the 3rd and 4th cadmium level increased, suggesting that patients with osteoporosis exhibiting higher cadmium levels tended to utilize medical care more, potentially decreasing T-scores. Such findings further indicate that patients with osteoporosis with higher cadmium levels may be relatively aware of their risk, although this hypothesis is difficult to verify using the current data. Furthermore, the OR at the highest (4th) level was still lower than that at the 3rd level.

Second, our results may have also been influenced by selection bias. The target population of the KNHANES only includes non-institutionalized citizens, meaning that patients with severe comorbidities may have been excluded from the survey. For example, cadmium is a known risk factor for stroke [34] and cancer [16], both of which increase the risk of osteoporosis [35, 36]. Patients experiencing long-term admission due to these diseases may have been excluded, which in turn may have resulted in selection bias at the highest cadmium level. In our subregion analysis, negative associations were observed for the femoral neck, indicating that selection bias may have occurred with regard to patients with femoral neck osteoporosis.

There were various results with the additional sensitivity analysis. According to our quintile analysis, there was a dose-response relationship until the 4th quintile, although it disappeared in the 5th quintile. Assuming that selection bias caused the dose-response relationship to disappear, this supports the hypothesis that it may have occurred in groups with high cadmium exposure. In addition, a weak dose-response relationship was observed in analyses performed using a T-score less than or equal to −2.0 (defined by mitigating the criteria for abnormal status). If selection bias occurred among individuals with osteoporosis in the higher cadmium exposure, it is possible that this approach has alleviated this problem to some extent.

Blood cadmium levels were relatively lower among our patients than among those in previous studies [5, 37]. This discrepancy may be due to the inclusion of a nationally representative population in our study, in contrast to previous studies, which have focused on populations living in polluted regions. However, the risk of osteoporosis was noted even at low levels of cadmium exposure [7]. Furthermore, the benchmark dose lower bound (BMDL) of blood cadmium for osteoporosis for Chinese population has been reported as 1.39 μg/L [38], which is lower than the mean of the 3rd quartile in our study (1.59 μg/L). This may explain the sharp increase in the OR at the third quartile in our study.

Cadmium exposure can lead to decreases in BMD via several mechanisms. The major mechanisms involve renal tubular damage and interference with the metabolism of PTH and vitamin D [2, 3, 39]. However, we were unable to investigate these mechanisms in the present study. First, causal mediation analysis [40] is currently unavailable for use with multinomial models. Furthermore, a previous KNHANES revealed that serum creatinine, which is used to calculate the glomerular filtration rate (GFR) and as an index of renal function itself, is positively associated with BMD in healthy populations [41]. Therefore, it is difficult to determine the appropriate mediators for the model.

The strength of our study is that we demonstrated the possibility of an association between cadmium exposure and bone health in Korean post-menopausal women by complementing previous study using various methods. In addition to increasing sample size, utilizing multiple imputations and multinomial models, and adjusting weights given the complex survey design, we considered causal mechanisms in constructing our model. The disjunctive cause criterion is recommended for confounder selection when one cannot know all causal mechanisms related to exposures or outcomes [18]. Bias can occur if the cause of a variable is cadmium exposure (e.g., cancer [16], hypertension [42], or kidney function [2, 39]) or osteoporosis (e.g., fracture or osteoporosis medication) and the variable is controlled via exclusion or adjustment. Therefore, we only considered confounders that were the cause of exposure, outcomes, or both.

There are additional limitations in our study. First, blood cadmium level was used as index of cadmium exposure. However, blood cadmium is predominantly influenced by recent cadmium exposure. Urinary cadmium levels may therefore be more appropriate for investigating osteoporosis given their long-term stability [43]. However, the KNHANES did not assess urinary cadmium levels. Furthermore, blood and urinary cadmium levels are highly correlated [44], and several previous studies have utilized blood cadmium levels to investigate changes in BMD [5, 37, 45]. Moreover, cadmium excretion can occur when patients experience tubular proteinuria, which may make measurement of blood cadmium levels more appropriate [46]. Second, there may have been issues with confounder selection due to the use of cross-sectional data. For example, serum 25(OH)D3 level was used as an index of vitamin D deficiency. If the conversion of 25(OH)D3 to calcitriol is disturbed by cadmium, serum levels of 25(OH)D3 may be affected [2]. In this case, cadmium acts as a mediator, rather than a confounder [14]. Furthermore, it is possible that patients underwent hormone replacement therapy due to cadmium-induced osteoporosis caused by cadmium. Controlling for this variable would thus induce collider bias [18]. Further prospective studies are required to provide non-biased estimates.

## Conclusion

Our findings demonstrate that there may be a positive association between cadmium levels and the risk of osteopenia and osteoporosis in Korean menopausal women. However, further prospective studies are required to explain the absence of a dose-response relationship and address potential selection bias.

## Supplementary information

ESM 1 (DOCX 285 kb)

ESM 2 (DOCX 20 kb)

ESM 3 (DOCX 20 kb)

ESM 4 (DOCX 24 kb)

## Data Availability

KNHANES data were provided by the Korea Centers for Disease Control and Prevention. Researchers can freely access KNHANES data using the following link: https://knhanes.cdc.go.kr/knhanes/main.do.

## Code availability

R studio (version 1.1.463) was used for analysis.
