# Supplementary material for: Association between blood cadmium levels and the risk of osteopenia and osteoporosis in Korean post-menopausal women
Source: Arch Osteoporos. 2021 Feb 2;16(1):22. doi: 10.1007/s11657-021-00887-9 (PMC7850996; doi:10.1007/s11657-021-00887-9)
Supplement: Supplementary file 1 — (DOCX 285 kb) [file 11657_2021_887_MOESM1_ESM.docx]

**Association between blood cadmium levels and the risk of osteopenia and osteoporosis in Korean post-menopausal women**

Osteoporosis International

Eun-San Kim ^a^, Sangah Shin ^b^, Yoon Jae Lee ^a^, In-Hyuk Ha ^a^*

^a^ Jaseng Spine and Joint Research Institute, Jaseng Medical Foundation, 3F, 538 Gangnam-daero, Gangnam-gu, Seoul 06110, Republic of Korea

^b^ Department of Food and Nutrition, Chung-Ang University, Gyeonggi-do 17546, Republic of Korea

**Corresponding author:** In-Hyuk Ha

Jaseng Spine and Joint Research Institute, Jaseng Medical Foundation, 3F, 538 Gangnam-daero, Gangnam-gu, Seoul 06110, Republic of Korea

E-mail: [hanihata@gmail.com](mailto:hanihata@gmail.com)

ORCID: http://orcid.org/ 0000-0002-5020-6723

**Online Resource 1. A directed acyclic graph (DAG) representing the causal relationships surrounding exposure and outcomes.**


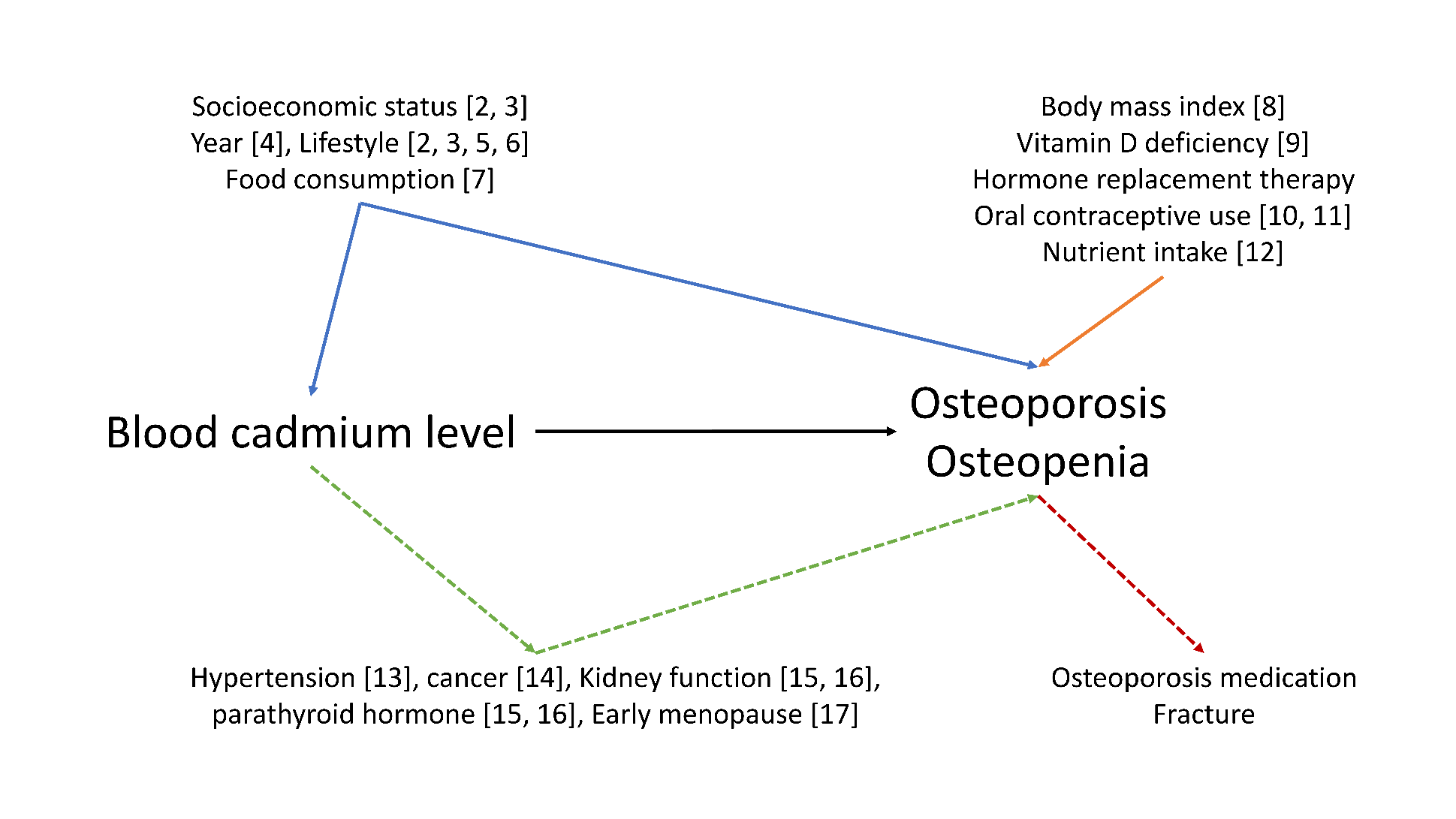


The criteria for confounder selection are presented using a directed acyclic graph (DAG). The variable in the tail of each arrow represents the cause of the variable at the head of the arrow. To minimize confounding bias, we selected confounders based on the disjunctive cause criterion [1], which controls the causes of the exposure, outcome, or both. When the outcome of exposure is controlled in cases of mediators or colliders, bias can occur. Causal relationships were determined based on the results of previous studies. A solid line represents an association that must be controlled, while a dashed line represents an association for which control would result in bias. The black line indicates the causal effects of exposure on outcome. Blue lines indicate confounding effects on exposure and outcome, which must be controlled using the back-door criterion [2-7]. The orange line indicates effects on the outcome only, which was controlled by the disjunctive cause criterion [8-12]. Green lines indicate indirect effects, which mediate the effect of exposure [13-17]. The red line indicates the outcome of osteoporosis or osteopenia.

**References**

[1] T.J. VanderWeele, Principles of confounder selection, European journal of epidemiology 34(3) (2019) 211-219.

[2] L. Järup, M. Berglund, C.G. Elinder, G. Nordberg, M. Vanter, Health effects of cadmium exposure–a review of the literature and a risk estimate, Scandinavian journal of work, environment & health (1998) 1-51.

[3] J. Tyrrell, D. Melzer, W. Henley, T.S. Galloway, N.J. Osborne, Associations between socioeconomic status and environmental toxicant concentrations in adults in the USA: NHANES 2001–2010, Environment international 59 (2013) 328-335.

[4] J.-W. Seo, B.-G. Kim, Y.-M. Kim, R.-B. Kim, J.-Y. Chung, K.-M. Lee, Y.-S. Hong, Trend of blood lead, mercury, and cadmium levels in Korean population: data analysis of the Korea National Health and Nutrition Examination Survey, Environmental monitoring and assessment 187(3) (2015) 146.

[5] H.-D. Jang, J.-Y. Hong, K. Han, J.C. Lee, B.-J. Shin, S.-W. Choi, S.-W. Suh, J.-H. Yang, S.-Y. Park, C. Bang, Relationship between bone mineral density and alcohol intake: A nationwide health survey analysis of postmenopausal women, PLoS One 12(6) (2017) e0180132.

[6] S.Y. Jung, S. Kim, K. Lee, J.Y. Kim, W.K. Bae, K. Lee, J.-S. Han, S. Kim, Association between secondhand smoke exposure and blood lead and cadmium concentration in community dwelling women: the fifth Korea National Health and Nutrition Examination Survey (2010–2012), BMJ open 5(7) (2015).

[7] H.-S. Lee, Y.-H. Cho, S.-O. Park, S.-H. Kye, B.-H. Kim, T.-S. Hahm, M. Kim, J.O. Lee, C.-i. Kim, Dietary exposure of the Korean population to arsenic, cadmium, lead and mercury, Journal of Food Composition and Analysis 19 (2006) S31-S37.

[8] G. Barrera, D. Bunout, V. Gattás, M.P. de la Maza, L. Leiva, S. Hirsch, A high body mass index protects against femoral neck osteoporosis in healthy elderly subjects, Nutrition 20(9) (2004) 769-771.

[9] M.F. Holick, Vitamin D: importance in the prevention of cancers, type 1 diabetes, heart disease, and osteoporosis, The American journal of clinical nutrition 79(3) (2004) 362-371.

[10] S. Liu, C. Lebrun, Effect of oral contraceptives and hormone replacement therapy on bone mineral density in premenopausal and perimenopausal women: a systematic review, British journal of sports medicine 40(1) (2006) 11-24.

[11] Y.Z. Bagger, L.B. Tankó, P. Alexandersen, H.B. Hansen, A. Møllgaard, P. Ravn, P. Qvist, J.A. Kanis, C. Christiansen, Two to three years of hormone replacement treatment in healthy women have long-term preventive effects on bone mass and osteoporotic fractures: the PERF study, Bone 34(4) (2004) 728-735.

[12] E. Warensjö, L. Byberg, H. Melhus, R. Gedeborg, H. Mallmin, A. Wolk, K. Michaëlsson, Dietary calcium intake and risk of fracture and osteoporosis: prospective longitudinal cohort study, Bmj 342 (2011) d1473.

[13] M. Tellez-Plaza, A. Navas-Acien, C.M. Crainiceanu, E. Guallar, Cadmium exposure and hypertension in the 1999–2004 National Health and Nutrition Examination Survey (NHANES), Environmental health perspectives 116(1) (2008) 51-56.

[14] A. Hartwig, Cadmium and cancer, Cadmium: From Toxicity to Essentiality, Springer2013, pp. 491-507.

[15] G. Kazantzis, Cadmium, osteoporosis and calcium metabolism, Biometals 17(5) (2004) 493-498.

[16] E.R. Youness, N.A. Mohammed, F.A. Morsy, Cadmium impact and osteoporosis: mechanism of action, Toxicology Mechanisms and Methods 22(7) (2012) 560-567.

[17] X. Chen, G. Zhu, T. Jin, Effects of cadmium exposure on age of menarche and menopause, Toxics 6(1) (2018) 6.
