# Supplementary material for: Association between blood cadmium levels and the risk of osteopenia and osteoporosis in Korean post-menopausal women
Source: Arch Osteoporos. 2021 Feb 2;16(1):22. doi: 10.1007/s11657-021-00887-9 (PMC7850996; doi:10.1007/s11657-021-00887-9)
Supplement: Supplementary file 2 — (DOCX 20 kb) [file 11657_2021_887_MOESM2_ESM.docx]

**Association between blood cadmium levels and the risk of osteopenia and osteoporosis in Korean post-menopausal women**

Osteoporosis International

Eun-San Kim ^a^, Sangah Shin ^b^, Yoon Jae Lee ^a^, In-Hyuk Ha ^a^*

^a^ Jaseng Spine and Joint Research Institute, Jaseng Medical Foundation, 3F, 538 Gangnam-daero, Gangnam-gu, Seoul 06110, Republic of Korea

^b^ Department of Food and Nutrition, Chung-Ang University, Gyeonggi-do 17546, Republic of Korea

**Corresponding author:** In-Hyuk Ha

Jaseng Spine and Joint Research Institute, Jaseng Medical Foundation, 3F, 538 Gangnam-daero, Gangnam-gu, Seoul 06110, Republic of Korea

E-mail: [hanihata@gmail.com](mailto:hanihata@gmail.com)

ORCID: http://orcid.org/ 0000-0002-5020-6723

**Online Resource 2. Additional analysis with different blood cadmium level categorization**

|  | Osteopenia | | | Osteoporosis | | |
| --- | --- | --- | --- | --- | --- | --- |
| BCd level | Prevalence | Unadjusted | Adjusted | Prevalence | Unadjusted | Adjusted |
| First quintile | 50.94 (41.84 - 60.05) | Ref | Ref | 33.47 (25.04 - 41.89) | Ref | Ref |
| Second quintile | 46.94 (37.67 - 56.21) | 0.92 (0.71 - 1.19) | 0.99 (0.70 - 1.40) | 37.34 (27.47 - 47.22) | 1.14 (0.84 - 1.54) | 1.33 (0.83 - 2.12) |
| Third quintile | 58.29 (48.86 - 67.72) | 2.03 (1.49 - 2.77) | 2.45 (1.49 - 4.01) | 32.77 (23.64 - 41.91) | 1.80 (1.25 - 2.58) | 2.42 (1.31 - 4.49) |
| Fourth quintile | 46.99 (37.27 - 56.72) | 1.32 (0.96 - 1.83) | 1.69 (1.02 - 2.82) | 42.45 (32.88 - 52.01) | 1.88 (1.37 - 2.59) | 2.83 (1.48 - 5.40) |
| Fifth quintile | 44.95 (35.26 - 54.64) | 1.03 (0.80 - 1.33) | 1.20 (0.75 - 1.93) | 41.77 (31.46 - 52.07) | 1.49 (1.12 - 1.98) | 1.66 (0.86 - 3.19) |
| P for trend | ━ | ━ | <0.001 | ━ | ━ | <0.001 |
| The blood cadmium exposure was categorized into quintile (five sections). The prevalence rates and odds ratios (ORs) are presented with 95% confidence intervals for each quartile. BCd: Blood cadmium | | | | | | |
