# Supplementary material for: Association between blood cadmium levels and the risk of osteopenia and osteoporosis in Korean post-menopausal women
Source: Arch Osteoporos. 2021 Feb 2;16(1):22. doi: 10.1007/s11657-021-00887-9 (PMC7850996; doi:10.1007/s11657-021-00887-9)
Supplement: Supplementary file 3 — (DOCX 20 kb) [file 11657_2021_887_MOESM3_ESM.docx]

**Association between blood cadmium levels and the risk of osteopenia and osteoporosis in Korean post-menopausal women**

Osteoporosis International

Eun-San Kim ^a^, Sangah Shin ^b^, Yoon Jae Lee ^a^, In-Hyuk Ha ^a^*

^a^ Jaseng Spine and Joint Research Institute, Jaseng Medical Foundation, 3F, 538 Gangnam-daero, Gangnam-gu, Seoul 06110, Republic of Korea

^b^ Department of Food and Nutrition, Chung-Ang University, Gyeonggi-do 17546, Republic of Korea

**Corresponding author:** In-Hyuk Ha

Jaseng Spine and Joint Research Institute, Jaseng Medical Foundation, 3F, 538 Gangnam-daero, Gangnam-gu, Seoul 06110, Republic of Korea

E-mail: [hanihata@gmail.com](mailto:hanihata@gmail.com)

ORCID: http://orcid.org/ 0000-0002-5020-6723

**Online Resource 3. Additional analysis with different T-score criteria**

| BCd level | Prevalence | Unadjusted | Adjusted |
| --- | --- | --- | --- |
| **T score ≤ -1.8** |  |  |  |
| First quartile | 61.79 (54.52 - 69.06) | Ref | Ref |
| Second quartile | 60.31 (52.64 - 67.98) | 0.94 (0.61 - 1.44) | 0.89 (0.51 - 1.56) |
| Third quartile | 67.49 (60.61 - 74.37) | 1.28 (0.83 - 1.98) | 1.53 (0.91 - 2.57) |
| Fourth quartile | 70.55 (63.50 - 77.61) | 1.48 (0.93 - 2.37) | 1.56 (0.87 - 2.79) |
| P for trend | ━ | ━ | 0.050 |
| **T score ≤ -2.0** |  |  |  |
| First quartile | 51.12 (43.47 - 58.77) | Ref | Ref |
| Second quartile | 56.50 (48.57 - 64.42) | 1.24 (0.80 - 1.93) | 1.21 (0.71 - 2.05) |
| Third quartile | 58.35 (50.70 - 66.00) | 1.34 (0.87 - 2.07) | 1.60 (0.95 - 2.71) |
| Fourth quartile | 62.45 (54.72 - 70.18) | 1.59 (1.01 - 2.51) | 1.76 (1.02 - 3.05) |
| P for trend | ━ | ━ | 0.027 |
| The outcome was defined with other T-score criteria. The logistic regression was used. The prevalence rates and odds ratios (ORs) are presented with 95% confidence intervals for each quartile. BCd: Blood cadmium. | | | |
