# Supplementary material for: Association between blood cadmium levels and the risk of osteopenia and osteoporosis in Korean post-menopausal women
Source: Arch Osteoporos. 2021 Feb 2;16(1):22. doi: 10.1007/s11657-021-00887-9 (PMC7850996; doi:10.1007/s11657-021-00887-9)
Supplement: Supplementary file 4 — (DOCX 24 kb) [file 11657_2021_887_MOESM4_ESM.docx]

**Association between blood cadmium levels and the risk of osteopenia and osteoporosis in Korean post-menopausal women**

Osteoporosis International

Eun-San Kim ^a^, Sangah Shin ^b^, Yoon Jae Lee ^a^, In-Hyuk Ha ^a^*

^a^ Jaseng Spine and Joint Research Institute, Jaseng Medical Foundation, 3F, 538 Gangnam-daero, Gangnam-gu, Seoul 06110, Republic of Korea

^b^ Department of Food and Nutrition, Chung-Ang University, Gyeonggi-do 17546, Republic of Korea

**Corresponding author:** In-Hyuk Ha

Jaseng Spine and Joint Research Institute, Jaseng Medical Foundation, 3F, 538 Gangnam-daero, Gangnam-gu, Seoul 06110, Republic of Korea

E-mail: [hanihata@gmail.com](mailto:hanihata@gmail.com)

ORCID: http://orcid.org/ 0000-0002-5020-6723

**Online Resource 4. Additional analysis with bone mineral density and T-score**

|  | BMD | | T-score | |
| --- | --- | --- | --- | --- |
| BCd level | Unadjusted | Adjusted | Unadjusted | Adjusted |
| **Total hip** |  |  |  |  |
| First quartile | Ref | Ref | Ref | Ref |
| Second quartile | -0.01 (-0.04 - 0.02) | -0.01 (-0.03 - 0.02) | -0.09 (-0.34 - 0.17) | -0.06 (-0.25 - 0.14) |
| Third quartile | -0.01 (-0.04 - 0.02) | -0.01 (-0.03 - 0.01) | -0.09 (-0.32 - 0.14) | -0.08 (-0.25 - 0.08) |
| Fourth quartile | 0.00 (-0.04 - 0.03) | 0.00 (-0.02 - 0.02) | -0.04 (-0.32 - 0.24) | 0.00 (-0.20 - 0.21) |
| P for trend |  | 0.906 |  | 0.906 |
| **Femoral neck** |  |  |  |  |
| First quartile | Ref | Ref | Ref | Ref |
| Second quartile | -0.01 (-0.03 - 0.02) | -0.01 (-0.02 - 0.01) | -0.07 (-0.30 - 0.17) | -0.05 (-0.23 - 0.13) |
| Third quartile | -0.01 (-0.03 - 0.01) | -0.01 (-0.02 - 0.01) | -0.08 (-0.28 - 0.13) | -0.07 (-0.23 - 0.09) |
| Fourth quartile | 0.00 (-0.02 - 0.03) | 0.01 (-0.01 - 0.03) | 0.02 (-0.23 - 0.27) | 0.08 (-0.10 - 0.27) |
| P for trend |  | 0.464 |  | 0.464 |
| **Lumbar spine** |  |  |  |  |
| First quartile | Ref | Ref | Ref | Ref |
| Second quartile | -0.01 (-0.04 - 0.02) | -0.01 (-0.04 - 0.02) | -0.10 (-0.38 - 0.18) | -0.08 (-0.33 - 0.17) |
| Third quartile | -0.02 (-0.05 - 0.01) | -0.02 (-0.05 - 0.01) | -0.18 (-0.46 - 0.10) | -0.19 (-0.45 - 0.07) |
| Fourth quartile | -0.02 (-0.05 - 0.02) | -0.01 (-0.04 - 0.02) | -0.15 (-0.46 - 0.15) | -0.09 (-0.36 - 0.18) |
| P for trend |  | 0.369 |  | 0.369 |
| **Lowest index** |  |  |  |  |
| First quartile | Ref | Ref | Ref | Ref |
| Second quartile | -0.01 (-0.03 - 0.02) | -0.01 (-0.03 - 0.01) | -0.09 (-0.33 - 0.14) | -0.06 (-0.25 - 0.12) |
| Third quartile | -0.01 (-0.03 - 0.01) | -0.01 (-0.03 - 0.01) | -0.17 (-0.38 - 0.04) | -0.17  (-0.34 - -0.01) |
| Fourth quartile | 0.00 (-0.02 - 0.03) | 0.01 (-0.01 - 0.03) | -0.11 (-0.35 - 0.13) | -0.04 (-0.23 - 0.15) |
| P for trend |  | 0.512 |  | 0.402 |
| The outcome was bone mineral density (BMD) and T-scores at each site. Moreover, the lowest BMD and T-score among sites were analyzed. The linear regression was used. The estimates are presented with 95% confidence intervals for each quartile. BCd: Blood cadmium; BMD: Bone mineral density | | | | |

The results with bone mineral density (BMD) and T-scores at each site was non-significant. However, the analysis with the lowest T-score among sites was significant (-0.17; 95% CI: -0.34 - -0.01).

We think these results are consistent with the context of the study, because osteoporosis is defined as having less than -2.5 T-score in any of the three sites. Depending on the age and comorbidity of the patient, BMD changes differently by each region. For example, BMD can 'increase' due to osteophyte caused by degenerative changes in the spine. So even if osteoporosis occurs in the femoral neck, BMD can be high when diagnosed only with the spine. Accordingly, the guidelines recommended measuring BMD in all sites and define osteoporosis in the case of having T-score less than -2.5 in any one place [1].

[1] N. Watts, J. Bilezikian, P. Camacho, S. Greenspan, S. Harris, S. Hodgson, M. Kleerekoper, M. Luckey, M. McClung, R. Pollack, American Association of Clinical Endocrinologists Medical Guidelines for Clinical Practice for the diagnosis and treatment of postmenopausal osteoporosis, Endocrine practice 16(Supplement 3) (2010) 1-37.
